# Microvascular Disease and Small-Vessel Disease: The Nexus of Multiple Diseases of Women

**DOI:** 10.1089/jwh.2019.7826

**Published:** 2020-06-10

**Authors:** Hena Patel, Neelum T. Aggarwal, Anupama Rao, Elizabeth Bryant, Rupa M. Sanghani, Mary Byrnes, Dinesh Kalra, Leigh Dairaghi, Lynne Braun, Sherine Gabriel, Annabelle Santos Volgman

**Affiliations:** ^1^ Department of Cardiology, Rush Medical College, Rush University, Chicago, Illinois.; ^2^ Department of Neurological Sciences, Rush Alzheimer's Disease Center, Rush Medical College, Rush University, Chicago, Illinois.; ^3^ Rush Medical College, Rush University, Chicago, Illinois.; ^4^ Clinical Nursing, Rush Medical College, Rush University, Chicago, Illinois.; ^5^ Rush College of Nursing and Medicine, Rush University, Chicago, Illinois.; ^6^ Department of Rheumatology, Rush Medical College, Rush University, Chicago, Illinois.

**Keywords:** small-vessel disease, microvascular disease, microvascular dysfunction, coronary perfusion reserve, women, cardiovascular disease

## Abstract

Microvascular disease, or small-vessel disease, is a multisystem disorder with a common pathophysiological basis that differentially affects various organs in some patients. The prevalence of small-vessel disease in the heart has been found to be higher in women compared with men. Additionally, other diseases prominently affecting women, including heart failure with preserved ejection fraction, Takotsubo cardiomyopathy, cerebral small-vessel disease, preeclampsia, pulmonary arterial hypertension (PAH), endothelial dysfunction in diabetes, diabetic cardiomyopathy, rheumatoid arthritis, systemic lupus erythematosus, and systemic sclerosis, may have a common etiologic linkage related to microvascular disease. To the best of our knowledge this is the first article to investigate this potential linkage. We sought to identify various diseases with a shared pathophysiology involving microvascular/endothelial dysfunction that primarily affect women, and their potential implications for disease management. Advanced imaging technologies, such as magnetic resonance imaging and positron-emission tomography, enable the detection and increased understanding of microvascular dysfunction in various diseases. Therapies that improve endothelial function, such as those used in PAH, may also be associated with benefits across the full spectrum of microvascular dysfunction. A shared pathology across multiple organ systems highlights the need for a collaborative, multidisciplinary approach among medical subspecialty practitioners who care for women with small-vessel disease. Such an approach may lead to accelerated research in diseases that affect women and their quality of life.

## Introduction

Ischemic heart disease, dementia, and stroke are leading causes of disability and death.^1^ Small-vessel disease, or microvascular disease, refers to a group of pathological processes with various etiologies affecting the small arteries, arterioles, venules, and capillaries.^2^ Berry et al. recently proposed that small-vessel disease is a multisystem disorder with a common pathophysiological basis that differentially affects various organs.^3^

**FIG. 1.**
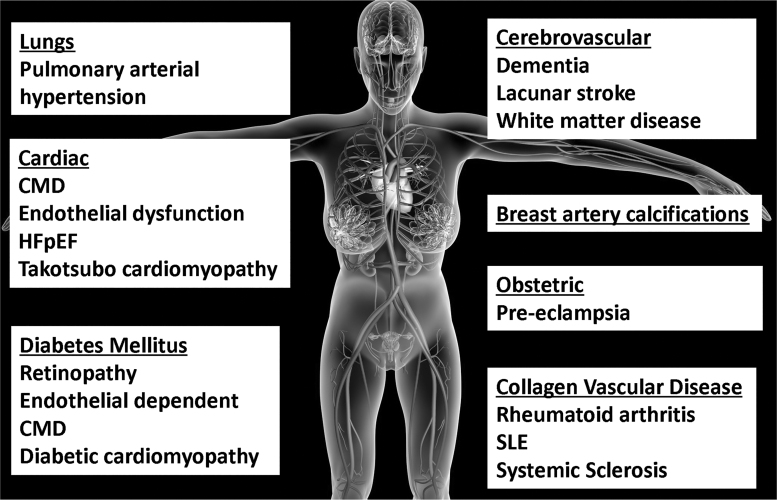
Small-vessel disease/microvascular disease affecting women more than men, a group of pathological process with various etiologies affecting the small arteries, arterioles, venules, and capillaries.

**FIG. 2.**
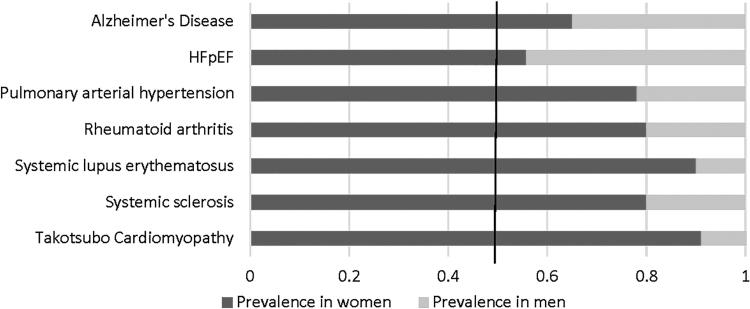
Disease prevalence in women versus men.

New imaging modalities, such as cardiac magnetic resonance imaging (CMR) and positron emission tomography (PET) scanning, suggest that this multisystem disorder may stem from altered endothelial cell function. The diseases included within this multisystem disorder are those that tend to predominantly affect women: coronary small-vessel disease^4^; cerebral small-vessel disease; preeclampsia; pulmonary arterial hypertension (PAH); diabetic cardiomyopathy; and some collagen vascular diseases (Figs. 1 and 2).

The potential connection between small-vessel disease in the heart and other organ systems may account for the higher prevalence in women. Alteration of endothelial cells is a hallmark of these diseases. New imaging modalities such as CMR and PET scanning have improved the understanding of endothelial function. Recognizing a shared pathology is especially important because the prevention and management of these various manifestations of microvascular dysfunction may also be similar. We sought to identify various diseases with a shared pathophysiology of microvascular/endothelial dysfunction and elucidate their potential implications for management.

## Endothelial Dysfunction

The endothelium plays a key role in vascular homeostasis through the release of a variety of autocrine and paracrine substances.^5^ In addition to vasoregulation, the endothelium inhibits platelet aggregation and adhesion, smooth muscle cell proliferation, and leukocyte adhesion. Endothelial dysfunction leads to a reversible shift in the properties of the endothelium toward reduced vasodilation, a proinflammatory state, and proliferative and prothrombotic properties. Endothelial dysfunction is an early event in atherogenesis and contributes to all the stages of atherosclerosis.^5^

Women may have several risk factors for endothelial dysfunction. Postmenopausal women may be at risk for endothelial dysfunction due to a decline in estrogen production. The beneficial effects of estrogen on improving flow through the coronary microcirculation through endothelium-dependent and independent mechanisms have been well described.^2^ Decreased estrogen levels during menopause are believed to increase sympathetic activation and endothelial dysfunction. Additionally, there is some evidence that psychosocial stressors and certain responses to them, such as depression and anxiety, may contribute to endothelial dysfunction and damage. Women are more likely to report chronic stress exposure, depression, and anxiety.^6^ The mechanisms behind these associations are still being studied.

## Cardiac Dysfunction

### Coronary microvascular disease

Coronary artery disease (CAD) is the leading cause of mortality in women with differing patterns of coronary atherosclerosis and extensive comorbidities compared with men.^7^ As many as 50% of women presenting with symptoms of angina have minimal or no angiographic CAD^7^ and assured that they have no CAD. Data from the Women's Ischemia Syndrome Evaluation (WISE) study suggest that patients with ischemia with nonobstructive coronary arteries (INOCA), compared with patients without persistent angina, are at a higher risk of repeat hospital admissions, increased rates of progression to obstructive CAD, and greater overall cardiovascular mortality and morbidity.^8^

Similarly, myocardial infarction with nonobstructive coronary arteries, or MINOCA (<50% stenosis), is more common among younger patients and women.^9^ The Acute Coronary Treatment and Intervention Outcomes Network Registry from 2007 to 2014 demonstrated that MINOCA was more common in women than men (10.5% vs. 3.4%; *p* < 0.0001).^9^ In fact, women may present with an entirely different pattern of ischemic heart disease from men, which includes a higher prevalence of angina but with lower obstructive CAD burden, as well as a poorer prognosis.^6^

This presence of angina with minimal or no angiographic CAD is referred to as coronary microvascular dysfunction (CMD). CMD can occur in both sexes, but is more prevalent in women, especially after menopause.^10^ Estimates from the WISE database show that there are at least three to four million Americans with ischemia despite the absence of obstructive atherosclerosis, with associated poor quality of life, psychological distress, and health-care costs that approximate those with obstructive CAD. Furthermore, microvascular disease is associated with a 2.5% annual major adverse cardiovascular event (MACE) rate.^11^

Studies involving CMD patients have revealed a number of underlying pathophysiological mechanisms, including endothelial dysfunction, reduced coronary flow reserve (CFR), and autonomic imbalance.^7^ Specifically, an imbalance between the endothelium-derived vasodilator nitric oxide (NO) and the vasoconstrictor endothelin-1 (ET-1) is a proposed mechanism for CMD.^10^

The Coronary Microvascular Angina (CorMicA) study tested the hypothesis that patients with INOCA also have functional abnormalities in the peripheral small arteries.^12^ Using arterioles from gluteal biopsies, patients with microvascular and vasospastic angina were found to have peripheral microvascular abnormalities characterized by reduced maximum relaxation following incubation with acetylcholine and increased responses to vasoconstrictor stimuli. This study demonstrated an association between CMD and small-vessel disease in other organs, including the brain and kidney. Thus, selective blockage of vascular endothelin receptors may be a promising new approach for the treatment of CMD and other diseases.

### Heart failure with preserved ejection fraction

Patients with heart failure with preserved ejection fraction (HFpEF) experience similar rates of morbidity and functional decline as do those with heart failure and reduced ejection fraction (HFrEF),^13^ but few effective treatments are available. Clinical studies suggest higher prevalence of HFpEF in women compared with men.^14^ Increasing evidence indicates a central role for endothelial dysfunction driven by comorbidities, such as hypertension, which contribute to the pathogenesis of HFpEF, leading to cardiomyocyte dysfunction, left ventricular concentric remodeling, and diastolic dysfunction.^13^

ET-1 synthesis in cardiac myocytes is increased during the hypertrophic response to hypertension and causes abnormalities in endothelial function, vascular compliance, diastolic relaxation, and myocardial fibrosis. Conversely, in HFrEF, direct cardiomyocyte injury is the predominant trigger for systemic neuroendocrine activation, left ventricular eccentric remodeling, and systolic dysfunction.^13^

Studies investigating ET-1 inhibitors in patients with HFpEF, have shown decreased left ventricular hypertrophy and remodeling, although with small sample sizes.^15^ A large study evaluated the use of valsartan and sacubitril, a neprolysin inhibitor (a vasoactive peptide inhibitor) in patients with heart failure and an ejection fraction of 45% or higher. The results showed no significant decrease in the rate of total hospitalizations for heart failure and death from cardiovascular causes: risk ratio 0.87 (95% confidence interval [0.75–1.01]). However, subgroup analysis showed significant decrease in outcomes in women: risk ratio 0.73 (95% confidence interval [0.59–0.90]).^16^

### Takotsubo syndrome

There is a preponderance in the number of women with Takotsubo Syndrome (TTS) (women to men ratio 9:1, see Figure 2), especially postmenopausal women.^17^ Recent studies have highlighted the role of endothelial dysfunction leading to epicardial or microvascular coronary artery spasm as a pathogenic mechanism in TTS.^18^ The neurohormonal cascade triggered by acute stress may cause an imbalance between vasoconstricting and vasodilating factors, resulting in the transient myocardial ischemia and stunning typically observed in TTS.^18^

Endothelial dysfunction also may explain why TTS is more common in postmenopausal women, as they produce less estrogen than premenopausal women.^18^ The beneficial effects of estrogen on improving flow through the coronary microcirculation through endothelium-dependent and independent mechanisms has been well described.^19^ Decreased estrogen levels during menopause are believed to increase sympathetic activation and endothelial dysfunction.^18^ This critical link between estrogen and TTS was aptly demonstrated in an animal study where TTS was prevented by pretreatment with estrogen.^20^

Estrogen supplementation upregulated cardioprotective elements such as atrial natriuretic peptide and heat shock protein 70, in addition to attenuating the stress-induced hypothalamo-sympathoadrenal outflow from the central nervous system to the target organs.^20^ These emerging studies exposing the critical role of estrogen on the microcirculation provide further insights into the gender-specific differences in the incidence of TTS.

## Neurological Dysfunction

### Cerebral microvascular disease

Cerebral small-vessel disease can present clinically as stroke, microinfarct, or a cognitive syndrome.^3^ It encompasses a range of vascular pathologies, including arteriosclerosis, small-vessel atheroma, and cerebral amyloid angiopathy,^3^ and is the vascular source of cognitive impairment and dementia seen more commonly in women versus men.^21^

Neuropathological and brain magnetic resonance imaging (MRI) studies suggest that decreased vascular density occurs with aging and Alzheimer's disease, and these changes precede the onset of cognitive dysfunction and neurodegeneration.^22^ There is also a decline in cerebrovascular angiogenesis, which may inhibit recovery from hypoxia-induced capillary loss. Cerebral blood flow may then be further inhibited by tortuous arterioles and deposition of excessive collagen in the veins and venules.

Loss of perfusion due to capillary loss appears to precede cell loss in leukoaraiosis, and cerebral blood flow is also reduced in the apparently normal white matter. In patients with Alzheimer's disease, chronic cerebral hypoperfusion and glucose hypometabolism may occur for many decades before cognitive decline is clinically evident. This hypoperfusion may induce white matter lesions, which are commonly seen in brain MRI. Other MRI markers of subclinical vascular brain disease—microinfarcts, microbleeds, demyelinization, and axonal damage—are strongly associated with atherosclerotic calcifications, and are increasingly seen in people with Alzheimer's disease.

Recent data have shown that two-thirds of patients diagnosed with dementia due to Alzheimer's disease are women.^23^ The ways in which coronary microvascular disease impacts expression of Alzheimer's disease in women is an area of intense interest. These conditions may share and synergistically activate inflammation that upregulates cerebrovascular pathology through proinflammatory cytokines, ET-1, and NO.

## Pulmonary Dysfunction

### Pulmonary arterial hypertension

PAH is a cardiopulmonary disorder characterized by elevated pulmonary vascular resistance (PVR) and pulmonary artery pressure (PAP) leading to right-heart failure and death.^24^ PAH is a rare disease and predominantly targets women^24^ with a poor prognosis (3-year survival rate of 58%).^24^

Endothelial dysfunction and vascular remodeling cause progressive loss and ultimate obliteration of small pulmonary arteries, leading to the increased PVR and PAP seen in PAH.^25^ Current evidence strongly suggests a central role for endothelial dysfunction in the initiation and progression of PAH, thus therapies that improve the endothelial function or restore the altered balance of endothelium-derived vasoactive mediators are used to manage this disease. Similar therapies may be useful in CMD. A greater understanding of the role of the endothelium in PAH will facilitate the evolution of more advanced targeted therapies. Drugs, such as statins, angiotensin-converting enzyme inhibitors, antioxidants, and l-arginine supplementation reverse endothelial dysfunction in CAD^5^ and may well prove useful as adjunct therapies in PAH.

## Obstetric Dysfunction

### Preeclampsia

Preeclampsia affects ∼2%–8% of pregnancies worldwide, typically presenting after 20 weeks' gestation and resolved by 3 months postpartum.^26^ In preeclampsia, a failure of vascular remodeling in the maternal spiral arteries results in hypoperfusion, hypoxia, and constricted placental vessels.^27^ This causes a systemic inflammatory response, releasing various factors, including antiangiogenic proteins and inflammatory cytokines.^26^ Damaged endothelium causes generalized endothelial dysfunction in the peripheral, glomerular, and cerebral vessels, leading to hypertension, proteinuria, and seizures.^28^

In women with preeclampsia, vasoconstrictor ET-1 levels are increased.^29^ One study on microvascular dysfunction found that ET-1-mediated vasoconstriction was increased in the microvasculature of women with a history of preeclampsia.^30^ This may be attributed to the absence of ET-1 receptor type-B-mediated dilation, leading to an exaggerated vasoconstrictive response to ET-1.^30^

Flow-mediated dilation is also abnormal with preeclampsia. Chambers et al.^31^ compared women with healthy pregnancies to women with a history of preeclampsia, with a median interval of 3 years postpartum. Brachial artery flow-mediated dilation, measuring the diameter at end diastole, was lower in women with a history of preeclampsia than in those with healthy pregnancies. Because flow-mediated dilation is endothelium dependent, it points to impaired vascular endothelial function in women with a history of preeclampsia.^31^

Similarly, Yinon et al.^28^ recruited women 6 to 24 months after delivery, evaluating early onset preeclampsia (<34 weeks' gestation) and late-onset preeclampsia (≥34 weeks' gestation), and found that flow-mediated dilation was significantly reduced in women with previous early preeclampsia compared with women with previous late preeclampsia and control subjects.^28^ Although women with preeclampsia appear asymptomatic after delivering the placenta, an underlying dysfunction in the blood vessels remains.^30^

Women with a history of preeclampsia had a MACE in the 10 years following the birth in an affected pregnancy of 18.2% compared with 1.7% of women without preeclampsia.^32^ A recent meta-analysis comprising 22 studies and over six million women concluded that preeclampsia was associated with a fourfold increase in future incident heart failure and a twofold increased risk of MACE.^33^ Given the high risk of adverse cardiovascular disease (CVD) events in the years following preeclampsia, the 2018 Cholesterol Guidelines^34^ included preeclampsia as a risk-enhancing factor and incorporated in the clinician/patient risk discussion. The history of preeclampsia may encourage a clinician to recommend statin treatment to reduce CVD risk.

## Collagen Vascular Dysfunction

### Rheumatoid arthritis

Rheumatoid arthritis (RA) is a systemic inflammatory autoimmune disease associated with increased cardiovascular mortality and morbidity, and is considered an independent risk factor for early and accelerated atherosclerosis.^35^ The relative increase in the risk of myocardial infarction and stroke in RA patients compared with the general population is 68% and 41%, respectively.^36^ Furthermore, the increased risk of myocardial infarction is predominantly observed within the first 3 years following diagnosis, suggesting that the increased risk of CVD occurs in the early stages of the disease.^37^ Endothelial dysfunction, leading to accelerated atherogenesis, is suspected of playing a key role in the pathogenesis for the excess risk of CVD.^38,39^

There is no correlation between microvascular and macrovascular endothelial dysfunction in RA cohorts, suggesting a different underlying vascular pathology.^40^ Despite the increased incidence of CAD in RA, patients with RA frequently have myocardial ischemia in the absence of obstructive CAD, highlighting the influence of CMD.^41^ Such findings have important implications for the cardiovascular risk management of patients with RA, which is also a risk-enhancing factor in the 2018 Cholesterol Guidelines,^34^ along with other inflammatory conditions. Anti-inflammatory treatment for RA can improve microvascular endothelial function, supporting a pathogenic link between inflammation and microvascular dysfunction in this population.^42^

### Systemic lupus erythematosus

Systemic lupus erythematosus (SLE) is a multisystem autoimmune connective tissue disease primarily affecting young women. SLE carries a strong predisposition to CVD, including atherosclerosis, vascular inflammation, Raynaud's phenomenon, endothelial dysfunction, and a procoagulant tendency associated with antiphospholipid antibodies. Epidemiological studies estimate that the risk of myocardial infarction is increased by 5- to 10-fold in SLE patients compared with the general population,^43,44^ making it a significant risk factor for cardiovascular events. Furthermore, coronary death is a leading cause of long-term mortality several years after diagnosis.^45^ Standard risk factors cannot fully account for the increased risk of CAD in patients with SLE.^43^

In addition to obstructive CAD, chronic microvascular disease is also a common finding in SLE and is associated with worse cardiovascular outcomes.^46^ As in RA, microvascular dysfunction in SLE appears related to be both endothelial dysfunction^47^ and inflammatory status. Increased sympathetic outflow^48^ and increased levels of ET-1^49^ have been found in SLE patients. A reduced bioavailability of NO (due to either underproduction or increased destruction) in response to shear stress, along with increased oxidative stress, may also contribute to the difference in flow-mediated dilation.^50^ Inflammation is also proposed as an important trigger of endothelial damage, with type 1 interferon, along with other endothelial toxic mediators, playing a key role in mediating endothelial damage in patients with SLE.^51^

### Systemic sclerosis

Systemic sclerosis (SSc), or scleroderma, is a multisystem autoimmune connective tissue disease characterized by vascular dysfunction and fibrosis of the skin and internal organs.^52^ Raynaud's phenomenon, PAH, and scleroderma renal crisis are the main clinical presentations.^53^ The cause and pathogenesis of SSc remain uncharacterized; however microvascular endothelial cell dysfunction is a key feature of SSc. Microvascular involvement may predict CAD over time.^53^ Endothelial damage, triggered by infection-induced apoptosis, immunomediated cytotoxicity, anti-endothelial antibodies or ischemia/reperfusion injury, occurs in the early stages of SSc.^53^

Overproduction of endothelin and impaired NO and prostacyclin release, mediates vasospasm and contributes to intimal proliferation, vascular fibrosis, and stiffness of the vessel wall through the release of cytokines and profibrotic growth factors, including ET-1.^52,54^ Platelet activation and enhanced coagulation with reduced fibrinolysis lead to fibrin deposits, intimal proliferation, and luminal narrowing.^54^

Studies have shown a severe blunting of the CFR measured during cardiac catheterization in patients with cutaneous SSc with established myocardial involvement and normal coronary angiograms.^52^ Similarly, patients with asymptomatic scleroderma were shown to have a higher prevalence of reduced CFR. Early coronary microvascular involvement in patients with SSc has been linked to impaired prognosis.^52^

## Endocrine Dysfunction

### CMD in diabetics

Cardiovascular complications are the leading causes of diabetes-related morbidity and mortality and diabetes is a greater risk factor for cardiovascular mortality in women than in men.^55^ CMD is prevalent in diabetics and nonobstructive CAD. A study showed that patients with endothelial-dependent CMD were significantly older and more likely to be female than those without CMD.^56^ The study also showed that hemoglobin A1C and fasting serum glucose were significantly higher in women, but not in men with diabetes who had endothelial-independent and endothelial-dependent CMD. These findings suggest that there may be a link between glycemic control and functional coronary microvascular abnormalities in women, but not in men with diabetes, and that CMD may be a potential mediator of ischemia in diabetics with suboptimal glycemic control.^56^

Diabetic retinopathy, a major complication of diabetes mellitus, is recognized as a microvascular disease. Hyperglycemia is thought to promote retinal microvascular damage through multiple metabolic pathways.^57^ Interestingly, there may be a link between retinal microvascular changes and microvascular angina. In a study of 915 participants who had both retinal photography and coronary angiography, narrower retinal venules were associated with microvascular angina in women but not in men. However, no differences were observed in retinal arterioles. Approximately 25% of participants with microvascular angina had a history of diabetes, and more women than men with microvascular angina had elevated hemoglobin A1C.^58^

### Diabetic cardiomyopathy

Diabetic cardiomyopathy is a microvascular disease that can lead to heart failure and occurs independently of CAD. The pathogenesis of diabetic cardiomyopathy involves vascular endothelial cell dysfunction, as well as myocyte necrosis. It is characterized by interstitial fibrosis and impaired myocardial perfusion, suggesting microvascular disease.^59^ Vascular remodeling occurs as a result of longstanding hyperglycemia and impaired NO production, leading to increased production of glycated proteins and endothelial vascular growth factor.^60^ Other contributing metabolic abnormalities may include defective glucose transport, increased myocyte fatty acid uptake, and dysmetabolism. These biochemical changes manifest as hemodynamic alteration, capillary basement membrane thickening, interstitial fibrosis, and myocyte hypertrophy and necrosis.^61^

Renin antagonist drugs have been to protect against diabetic cardiomyopathy.^62,63^ Sildenafil, a selective phosphodiesterase type 5 inhibitor and vasodilator, was recently shown to improve myocardial function, cardiac remodeling, and some circulatory markers of cardiac inflammation in patients with diabetic cardiomyopathy.^64^ Trials are needed to study the role of sildenafil in diabetic cardiomyopathy.

## Imaging Modalities to Study Endothelial Dysfunction

### Breast arterial calcifications

Breast arterial calcifications (BAC) are benign sheet-like calcium deposits in the media of breast arteries that cause circumferential thickening resulting in stiffer and less compliant vessels.^65^ BAC may be an early marker of endothelial dysfunction and vascular stiffness and correlates with MACE.^66,67^ Furthermore, BAC are also associated with increased risk of CAD.^68^ Approximately 12.7% of screening mammograms have BAC and may be a cost-effective method of identifying women at risk for CMD.^66^

### PET perfusion imaging

Cardiac PET perfusion imaging with rubidium-82 or nitrogen-13 ammonia allows for absolute noninvasive quantification of myocardial blood flow. Physiologically, myocardial flow reserve (MFR) integrates the entire coronary circulation and represents flow reserve across this entire spectrum, with assessment of focal, diffuse, and coronary microcirculation, myocardial tissue perfusion, and microvascular dysfunction.^69^ MFR is an important prognostic risk indicator, and have shown that patients with low MFR have higher MACE rates.^70,71^ On adjusted analysis, MFR was found to be the primary driver of sex differences in risk who had the paradox of ischemia and symptoms, but normal coronary arteries.^72^

### Cardiac magnetic resonance imaging

Stress perfusion cardiac MRI is another ideal noninvasive method of assessing CMD. Invasive coronary reactivity testing (CRT) is the reference standard for the diagnosis of CMD. Abnormal CRT values are well established as adverse prognostic markers in patients with and without obstructive CAD.^73,74^ When compared with CRT, standard noninvasive tests such as stress echocardiography and myocardial single-photon computerized tomography imaging can often have normal results in patients with microvascular dysfunction.^75^ Stress cardiac MRI using a vasodilator such as adenosine or regadenoson, has been validated against CRT as a reliable diagnostic method, allowing for the detection of CMD with high spatial resolution but without the use of ionizing radiation.

Even though invasive CRT is considered the gold standard for evaluation of CMD and is generally safe in experienced centers,^11,76^ it is invasive and cannot be done on a serial basis to track progression or improvement after various treatments. Hence, noninvasive techniques using PET or CMR have become clinically very useful as they allow one to establish the diagnosis with good accuracy and track changes after treatment. Compared with PET, CMR has better spatial resolution (1.5 mm), wider scanner availability, and lacks ionizing radiation. Even though rest and stress coronary flow measurements have differed between CMR and PET studies, the ratio (CFR) is similar, because the variations in quantification affect stress and rest equally.

Since CFR can vary among normal individuals based on age and sex, the cutoffs using different techniques are different. In general, a CFR on PET of >2.5 is defined as the cutoff for normal and <1.5 as abnormal.^77^ However, CFR also carries important prognostic information in the gray zone of 1.5 to 2.5. CMR can assess myocardial perfusion based on changes in the myocardial T1-weighted signal intensity of gadolinium-based contrast agents which shorten the T1 relaxation time of myocardium. During the first pass, gadolinium contrast diffuses in the interstitial space from the microvasculature resulting in increased signal intensity proportional to the perfusion and blood volume of the myocardium, size of the extravascular compartment, and capillary permeability.

Time activity curves of the contrast agent as it perfuses the myocardium can be generated for both endocardial and mid myocardial layers using a conventional 16-segment model of the left ventricle and quantified to give signal intensity after vasodilator stress as well as at rest. The design of CMR perfusion pulse sequences and magnetic gradient strengths have improved rapidly over the past decade such that they now have high spatial and temporal resolution, linearity between signal intensity and contrast agent concentration, and high signal/noise ratio. In a seminal substudy of the WISE trial involving 118 women with CMD diagnosed by the reference standard of invasive CRT, myocardial perfusion reserve index (MPRI) was lower in symptomatic women as compared with 21 control subjects (1.7 vs. 2.2, *p* < 0.0001). An abnormal MPRI cutoff of <1.84 had a sensitivity of 73% and a specificity of 74% to identify an abnormal CRT.^5^

MRI perfusion reserve in patients with CMD also provides prognostic information and is linked to outcomes. In another substudy of the WISE trial, a model incorporating abnormal MRPI and EF was used to study 100 women who were then followed up for MACE over 42 months. Those women with a high risk score had an annualized MACE rate of 12% versus 4% in the group that was low risk.^78^

CMR perfusion can also distinguish between epicardial stenosis and CMD.^79^ In a recent study of 60 patients with angina, the magnitude of change in vasodilator stress T1 mapping could differentiate normals (ΔT1 ∼ 6%) versus those with CMD (ΔT1 ∼ 4%) or epicardial stenosis (ΔT1 < 1.5%). Other novel automated pixel-wide quantification techniques have also shown promise in small studies using adenosine stress MBF and have been validated against an IMR >25 (index of microvascular resistance) in the catheterization laboratory.^80^

The MRI substudy of the CorMicA trial in patients with angina, but without obstructive CAD will be the largest study to assess the association of quantitative myocardial perfusion by MRI with invasive coronary vascular function testing.^81^

### Invasive testing

CRT is an invasive procedure performed in a cardiac catheterization laboratory. It can evaluate the following independent pathways: (1) nonendothelial-dependent microvascular reactivity with intracoronary adenosine; (2) endothelial-dependent reactivity with intracoronary acetylcholine; and (3) nonendothelial-dependent epicardial coronary reactivity assessed with intracoronary nitroglycerin. Coronary artery reactivity testing has documented safety and efficacy, and is fairly easy to perform.^11,76^ It can offer significant additional clinical information in the woman with persistent angina and a normal angiogram, yet is underperformed and not widely available. Recent studies have shown that impaired coronary microvascular reactivity predicts MACE in women with signs and symptoms of ischemia, even in the absence of significant CAD.^82^

## Conclusion

Recent evidence suggests that small-vessel disease may be a multisystem disorder with shared mechanisms, clustering of vascular risk factors leading to an accelerated cardiovascular risk, and activation of the endothelin system affecting multiple organ systems.

It is essential that providers caring for women obtain a complete history with a focus on microvascular disorders, including a detailed pregnancy history, as standard of clinical care. Collecting a thorough history and integrating it when determining risk may be a better predictive model of long-term outcomes. CMD may be detected earlier than macrovascular disease with advanced imaging that can be used to guide diagnosis and preventive therapies. Since endothelial dysfunction correlates with cardiovascular risk and is reversible with interventions, it may be a useful selection criterion, target, and mechanistic surrogate endpoint in clinical trials regarding small-vessel diseases in women and men.

Therapies that improve endothelial function, such as statins, angiotensin-converting enzyme inhibitors, angiotensin receptor blockers, beta-adrenoceptor antagonists, or oral antidiabetic drugs may be beneficial across the full spectrum of microvascular dysfunction. Findings from ongoing studies, such as Women's Ischemia Treatment Reduces Events in Nonobstructive CAD (WARRIOR), a multicenter clinical trial evaluating impact of an aggressive treatment strategy for nonobstructive CAD versus traditional care to reduce the likelihood of major cardiovascular adverse events, are highly awaited.^83^

A shared pathology across multiple organ systems highlights the need for a collaborative, multidisciplinary approach among medical subspecialties caring for women with these diseases. Table 1 summarizes the potential research questions and clinical implications of the shared common pathway of endothelial dysfunction seen in various diseases afflicting women.

**Table 1.** Research Questions and Clinical Implications

Research questions
Do patients with documented CMD and endothelial dysfunction, seen with other diseases, benefit from aspirin, statins, or treatments that have an effect on the endothelium?
What is the role of female hormones in endothelial dysfunction? How can the sex differences in these diseases be explained?
Are there clinical, genetic, or epigenetic factors that trigger endothelial dysfunction in various microvascular beds?
Further research is needed to understand the exact mechanisms involved in the development and course of diabetic cardiomyopathy to facilitate the discovery of clinically effective targets for preventing this condition and its progression to heart failure.
What is the association of breast arterial calcification with endothelial dysfunction? Can it be used as a marker of CMD?

Clinical implications
Does the shared common pathway of endothelial dysfunction account for the increase in adverse cardiovascular events in these diseases?
Should patients with other diseases that involve endothelial dysfunction be referred to cardiovascular specialists?
What treatments and lifestyle changes can decrease the symptoms and adverse clinical outcomes of patients with endothelial dysfunction?

CMD, coronary microvascular dysfunction.
